# The effect of hybrid SCMC (BYOD) on foreign language anxiety and learning experience in comparison to pure SCMC and FTF communication

**DOI:** 10.3389/fpsyg.2023.1172442

**Published:** 2023-07-25

**Authors:** Xuecheng Liu

**Affiliations:** Nanjing University Libraries, Nanjing University, Nanjing, China

**Keywords:** SCMC, hybrid SCMC, BYOD, interaction in the language classroom, foreign language anxiety, second language acquisition, MDM technology

## Abstract

This study aims to investigate the impact of using synchronized computer-mediated communication (SCMC) in a face-to-face (FTF) classroom on reducing foreign language anxiety (FLA) and enhancing the learning experience. Fifty Chinese college students participated in a learning activity under three modes: normal FTF classroom (the blank sample), pure SCMC, and hybrid SCMC (BYOD). Smartphones, PCs, open internet, and the bring-your-own-device (BYOD) concept were used for SCMC applications. After completing the learning activity, the students completed Foreign Language Classroom Anxiety Scale (FLCAS) questionnaires. The students were also asked to complete perceptual questionnaires to assess their interaction, anxiety, distraction from the internet, and class atmosphere in the three modes. The results showed that the hybrid SCMC (BYOD) resulted in better interaction than the normal FTF classroom mode (the blank sample), while pure SCMC showed no significant improvement. Both SCMC modes reduced FLA compared to the normal FTF classroom mode (the blank sample), but pure SCMC caused a noticeable increase in distraction from the internet and weakened the classroom atmosphere. In contrast, the hybrid SCMC (BYOD) mode slightly increased distraction and improved the classroom atmosphere.

## 1. Introduction

Foreign language anxiety (FLA) is a prominent emotional factor that significantly influences second language acquisition, communication, thinking, and learning. FLA is notably rampant among Chinese college students, often leading to reluctance to participate and a resultant hindrance in interactive teaching.

Synchronous computer-mediated communication (SCMC) has been investigated as a potential strategy to alleviate FLA. Various modes, including text-SCMC, voice-SCMC, and virtual reality-SCMC (VR-SCMC), have been tested. Notably, VR-SCMC, a form of hybrid SCMC, integrates the advantages of both SCMC and face-to-face (FTF) modes. While certain studies have identified VR-SCMC as the most efficacious mode of learning, these same studies revealed little statistical difference in FLA reduction between hybrid and pure SCMC modes when measured using the FLCAS test. These findings prompt the conjecture that the positive learning experience associated with hybrid SCMC may derive not solely from diminished FLA but potentially from other factors inherent to the hybrid mode. Therefore, it becomes crucial to contemplate other pertinent factors, such as the learning atmosphere, to understand the impact of hybrid SCMC on the overall learning experience and interaction.

The transition to online teaching via pure SCMC in China during the COVID-19 pandemic has produced mixed outcomes regarding its effect on interaction and the learning environment. These observations, combined with earlier studies on hybrid SCMC, inspired this study’s hypothesis: hybrid SCMC, specifically when deployed as a supporting tool in a traditional FTF classroom under the Bring Your Own Device (BYOD) model, could potentially mitigate FLA and preserve a positive classroom atmosphere and effective teacher supervision. This unique blend of traditional and SCMC methods in a hybrid approach may enhance the overall learning experience and interaction. This study endeavors to test this hypothesis and enrich the field of classroom-based hybrid SCMC usage. Subsequent sections will delve deeper into previous studies and present a comprehensive literature review, providing additional context and corroborating evidence for our research.

## 2. Literature review

First, this section reviews existing studies on FLA in general and Chinese students’ FLA in particular to explain how detrimental FLA is to second language acquisition (SLA) and Chinese EFL learning. Then, previous laboratory studies on SCMC’s mitigating effect on FLA are discussed, especially York et al.’s (2021) conflicting result on hybrid SCMC (VR). Additionally, observations made by Chinese scholars about live webcast EFL teaching (pure SCMC) during Covid-19 period are also explored. These discussions collectively lead to the formulation of the hybrid SCMC (BYOD) mode hypothesis and the proposed research.

### 2.1. Previous studies on FLA and Chinese students’ FLA

Foreign language anxiety (FLA) has been identified as a significant emotional factor influencing second language (L2) learning. Students learning a second language may experience considerable anxiety, which can impede their ability to acquire the foreign language. Past research has shown that negative emotional reactions can interfere with the thinking and learning process (MacIntyre, 2017), consequently diminishing the effectiveness of even the best teaching methods and materials (Arnold and Brown, 1999). In recent decades, empirically tested methods, such as deep breathing, relaxing, soothing music (Oxford, 1990), instilling a positive belief in L2 learning (Oxford, 2017; Dewaele and MacIntyre, 2019; MacIntyre et al., 2019), and computer-mediated communication (CMC, York et al., 2021) have been implemented to reduce FLA. However, the results remain debatable, and no consensus has been reached on what should be changed in L2 learning (Toyama and Yamazaki, 2021) to relieve FLA. CMC, particularly that incorporating virtual reality (Deutschmann et al., 2009; Melchor-Couto, 2017; York et al., 2021), has been regarded as effective in reducing FLA. This important aspect merits further testing, research, and innovation.

Horwitz et al. (1986) developed the FLCAS (Foreign Language Classroom Anxiety Scale) to measure FLA. These scholars differentiated three primary distinct sources of language anxiety. First, communication apprehension represents the first source of language anxiety and is characterized by anxiety, shyness, or fear when communicating in a foreign language. Second, test anxiety denotes performance anxiety originating from the fear of failure in examinations. Finally, fear of negative evaluation is defined as “apprehension about others’ evaluation, avoidance of evaluative situations, and the expectation that others would evaluate oneself negatively” (Horwitz et al., 1986, pp. 127–128). FLCAS has become a widely used instrument to measure FLA since its first presentation.

Liu and Jackson (2008) used the Foreign Language Classroom Anxiety Scale (FLCAS) survey with Chinese EFL students. Results indicated prevalent Foreign Language Anxiety (FLA). In Liu and Jackson's (2008) study, over one third of 547 non-English major undergraduates felt classroom anxiety due to fear of negative evaluations (Liu and Jackson, 2008 Shao et al., 2013). Shao et al. (2013) further studied the learning anxiety and emotional intelligence of 510 Chinese students in Hangzhou, finding that about one third experienced high to moderate levels of anxiety in their English classes.

Li Yang’s “Crazy English” method, which uses techniques like shouting English and fostering positive beliefs, has successfully alleviated FLA in China. He gained significant popularity in China, Japan, and Korea and was appointed by the government for English training of Beijing Olympics 2008 volunteers. According to Lin (2010), a defining feature of Li Yang’s Crazy English is its slogan “enjoy losing face.” Its success underscores the importance of mitigating FLA in EFL instruction and highlights the severity of FLA within Confucian societies like China, Japan, and Korea.

Given that FLA has been shown to impede SLA and is prevalent among Chinese EFL students, who comprise the world’s largest group of SLA students, finding a method to alleviate FLA in Chinese EFL students is important. Therefore, research into the use of CMC as a promising means of reducing FLA in Chinese EFL is meaningful.

### 2.2. Previous SCMC experiments on reducing FLA

Computer-mediated communication (CMC) can be categorized into two types: asynchronous computer-mediated communication (ACMC) and synchronous computer-mediated communication (SCMC). ACMC refers to online communication that does not occur in real time. Examples include bulletin boards, Facebook comments, forums, and emails. SCMC allows communication to occur synchronously, with real-time voice, text, and even body language interaction. Commonly used SCMC applications include Skype, Webex, and Zoom.

In recent years, CMC, especially SCMC, has emerged as a novel approach to reducing FLA, which has been experimented with within language laboratories. Laboratory conditions involve special technological facilities, such as VR equipment, exclusive closed-circuit computer networks, special software, and specific tasks, designed and programmed especially for language research. These laboratory conditions are not used or available in most typical classroom teaching in the current world.

Warschauer (1996) found that speakers in SCMC mode demonstrated more equal participation than those in FTF mode due to a low threat of interaction. Charle Poza (2005) studied the effect of voice SCMC (voice board) and found that it reduced FLA by providing students with the opportunity to edit their vocal answers and reducing time pressure in the classroom. However, the study also revealed a negative attitude among participants toward visiting the language laboratory.

Satar and Özdener (2008) compared the effects of voice-SCMC mode and text-SCMC mode on FLA using a specifically created website. They found a significant reduction in FLA in the text group but not in the voice group. Satar and Özdener (2008) suggest that SCMC can effectively promote students’ confidence and create a safe environment for language practice and evaluation; 70% of participants in the text-SCMC group reported no worries about pronunciation, while only 20% in the voice group did. Satar and Özdener believed that the text-SCMC mode might have created a safer environment, leading to a more significant reduction in FLA.

Zhao and Lai (2009) proposed that the use of avatars in virtual world (VW)-based SCMC created a shielding effect, boosting students’ confidence and reducing anxiety. York et al. (2021) compared the effects of VR, voice, and video-based SCMC on FLA and found that VR was the most effective mode in reducing FLA and improving learning.

The abovementioned studies evaluated the ability of various SCMC modes to reduce FLA and identified possible causes for this reduction, such as editable answers, lower time pressure, a safer language learning environment, shielding effects, and increased confidence. However, these studies mainly used laboratory conditions. The impact of different SCMC modes, especially the hybrid mode, on FLA and learning in a typical classroom setting remains unknown, which serves as part of the motivation for this study. Here, SCMC is integrated into typical classroom teaching with inexpensive and widely available resources, including smartphones, open internet, PCs, normal school materials, and the BYOD concept.

The VR mode has garnered attention as a promising laboratory-tested hybrid SCMC mode. Gadelha (2018) believes that VR can potentially trigger an educational revolution due to its ability to provide students with an immersive experience in virtual environments. According to York et al. (2021), VR language communication in second and foreign language learning is similar to FTF communication. They consider VR-SCMC a hybrid mode that combines the benefits of FTF and SCMC communication.

Before conducting their three-mode experiment to compare the effects of VR, video, and voice SCMC on FLA, York et al. (2021) posed the following questions: Does VR, as a hybrid mode that offers paralinguistic cues and an immersive environment, improve comprehension and social presence and thus reduce FLA? Or does its decreased anonymity compared to video and voice modes increase anxiety instead?

York et al. (2021) conducted an experiment with 30 students to compare the impact of three SCMC modes on FLA: VR, voice, and video. The students were tasked with completing a “spot the difference” laboratory activity that allowed them to use body language and gestures in VR mode. Their FLA levels were then assessed in all three modes. Additionally, postexperiment questionnaires were given and open-ended responses were recorded to gather information about students’ explicit experiences and perceptions of the three modes.

The results of the experiment conducted by York et al. (2021) showed a reduction in FLA in all three modes; however, no significant difference in the reduction value between the modes was recorded. The postexperiment questionnaire and open-ended responses indicated a greater reduction in FLA in the VR mode than in the other two modes, with students mentioning that the VR mode was much more realistic, immersive, and fun. This discrepancy between the results of the FLCAS test and the postexperiment questionnaire raises questions regarding the validity of the results.

Two reasons were proposed by York et al. (2021) to explain this conflict: a low number of participants and participants’ curiosity about VR. However, these reasons do not competently explain the conflicting results. If a low number of participants was the cause, both the FLCAS questionnaire and perception questionnaire should have missed the difference between the modes. Additionally, participants’ curiosity about VR is not directly related to FLA, as defined by Horwitz et al. (1986). From the author’s perspective, such curiosity is more likely a positive affective factor that competes with FLA and is reflected in the perception questionnaire and open-ended responses but not in the FLCAS questionnaire. York’s focus on FLA may have led him to attribute all positive experiences to a decrease in FLA and thus find the results conflicting.

York’s conflicting result on hybrid SCMC (VR) leads the author to think that when studying the learning experience of hybrid SCMC (BYOD), the study should go beyond FLA and give attention to other possible affective factors as well.

### 2.3. Overview of live webcast teaching (pure SCMC) during the COVID-19 period in China

The COVID-19 pandemic led to a rapid expansion of live webcast-based SCMC teaching in China, home to the world’s largest population of L2 students. This shift provided Chinese teachers with the unique opportunity to observe the real-world impact of SCMC in EFL teaching. However, the observations were primarily confined to pure SCMC applications in online classrooms and lacked a theoretical connection to FLA and SCMC. There was also limited observation of hybrid SCMC application in the classroom, which forms part of the motivation for this study.

Pure SCMC offered several advantages over traditional face-to-face classroom interaction. With the shift to live webcasts (pure SCMC) in 2020 due to COVID-19 restrictions, two key observations emerged: (1) the live webcast platform provided teachers with various interaction modes, such as text and recorded audio, enabling the simultaneous receipt of multiple student queries and responses, thus reducing wait times and (2) students reported less anxiety and increased participation through SCMC, with some previously shy students becoming more active (Zhang, 2021). Additionally, the QQ live webcast platform created a less intimidating environment for students, effectively reducing their anxiety during English classes (Shen, 2020).

Live webcast teaching appeared to mitigate communication fears among students. In traditional foreign language teaching, shy students may not receive timely help due to classroom time constraints and teacher control to prevent distractions. Live webcasts, however, allowed students to directly interact with teachers, relieving pressure and encouraging active engagement (Liu, 2020).

Furthermore, SCMC teaching enabled one-to-one interaction between teachers and students, providing students with a more active classroom role (Li, 2021). The use of bullet comments, popular among college students for their entertainment value and high interactivity, satisfied their need for self-expression. In live webcast classes, students could more freely and positively complete exercises, respond to teachers’ questions, and express their opinions (Wei, 2020).

The live webcast (pure SCMC) mode of teaching has several limitations versus traditional face-to-face (FTF) teaching, such as a lack of supervision (Fu, 2020), difficulty in controlling the teaching atmosphere (Jin, 2020), and lack of learning atmosphere and consciousness (Qu et al., 2021). The absence of facial expressions, eye contact, and body language made it challenging to share emotions and information between teachers and students, leading to misinterpretations (Zhao, 2020). Poor supervision and weakened self-management skills among students were another issue (Yang, 2020). Additionally, students are easily distracted during online learning due to a lack of face-to-face interaction and supervision (Feng, 2020). These limitations were commonly noted by teachers and researchers during the pandemic period, highlighting the challenges of using the pure SCMC mode in practical teaching.

From the observations made by Chinese teachers during live webcast (pure SCMC online classroom) teaching in comparison with that in the traditional classroom (FTF), one advantage was identified: reduced fear and increased willingness to express. Two disadvantages were also noted: increased distractions and a weakened classroom atmosphere. These observations inform the author’s selection of affective factors for further study of the difference between hybrid and nonhybrid modes and for testing the hypothesis of the hybrid SCMC (BYOD) mode in section 2.4.

### 2.4. Research gap and the proposed study

This study seeks to address several research gaps within the realm of hybrid and nonhybrid SCMC in foreign language education:

1. Building upon York et al.’s (2021) investigation of hybrid SCMC (VR) and its mixed findings, this study aims to examine the impact of hybrid and nonhybrid SCMC on foreign language anxiety and potential differences in other affective factors.
2. Observations of purely SCMC-based webcast classes conducted by Chinese teachers have revealed two primary drawbacks: increased distractions due to limited supervision and a weaker classroom atmosphere. Consequently, this study explores the effects of two additional affective factors, classroom atmosphere and internet distractions, in the context of hybrid and nonhybrid SCMC.
3. Instead of using VR technology for the hybrid mode, this research opts for SCMC assistance in a face-to-face (FTF) classroom setting, employing personal computers, open internet access, smartphones, and the BYOD concept. This decision is based on three considerations:

- Studies focusing on classroom-based (BYOD) SCMC mode differences are scarce, and this research aims to contribute valuable insights into the pedagogical implications of hybrid and nonhybrid SCMC in a typical classroom environment.
- Utilizing SCMC through students’ personal devices and open internet access is a cost-effective and feasible solution, as language lab facilities and VR equipment may not be affordable for large-scale SLA teaching.
- The virtual reality experience differs from real-world FTF interactions, and conducting the study in a genuine FTF classroom setting allows the integration of real-world elements and benefits and the identification of potential weaknesses.

1. Finally, this study utilizes SCMC-assisted FTF classrooms for the hybrid mode while examining two additional affective factors alongside FLA. This study hypothesizes that SCMC assistance may reduce FLA, and the FTF classroom setting can maintain the positive classroom atmosphere typically associated with FTF modes and effective teacher supervision to prevent distractions. This combination could potentially result in an enhanced overall learning experience and interaction when compared to both the normal FTF mode and pure SCMC mode. The primary goal of this research is to test this hybrid SCMC (BYOD) mode hypothesis.

## 3. Research questions

1. Can using SCMC (BYOD) to assist learning in an FTF classroom effectively reduce learners’ FLA?
2. How does using SCMC (BYOD) to assist learning in an FTF classroom differ from pure SCMC and normal FTF classrooms regarding learning experience and interaction? Can the hypothesis suggesting a positive impact of the hybrid SCMC (BYOD) mode be supported?

## 4. Methodology

This study was motivated by the conflicting findings in York et al.’s (2021) experiment and Chinese scholars’ observations of pure SCMC practices. Building on the literature review, this study hypothesizes that integrating SCMC into a traditional face-to-face (FTF) classroom setting reduces students’ foreign language anxiety (FLA). Simultaneously, the FTF context maintains a classroom atmosphere and supervision to curb distractions typically found in traditional FTF classrooms, resulting in better interaction and learning experiences compared to both traditional FTF environments and pure SCMC online settings.

A self-contrast study was conducted with a single group of 50 students who participated in three learning modes: normal classroom mode (FTF) as the control group (blank sample), and pure SCMC and hybrid SCMC (BYOD) modes (SCMC-assisted FTF classroom) as the test groups. Students’ FLA scores and perceptual experiences were then assessed using questionnaires. As this was a self-contrast study with a single group of students, pretest surveys were not conducted to avoid any practice effects that might be introduced by repetitive testing. The normal classroom mode (FTF) served as the control group and blank sample, providing a baseline for the pretreatment situation.

### 4.1. Participants

This study included 50 s-year non-English major students (M age = 20.7, SD = 0.989) from a university in Nanjing, People’s Republic of China, comprising 27 females and 23 males. Each participant had passed the National Entrance Examination and was enrolled in a four-year, full-time undergraduate program. They were all taking a mandatory, credit-bearing English course designed for second-year non-English major students at the university. Despite having completed 11 years of compulsory English education, their English proficiency remained low. Only 30% of the participants passed the College English Test 4 (CET4, with a passing score of 425), which is the national and official college English test in China.

### 4.2. Instruments

#### 4.2.1. Learning activity observed

The learning activity observed was a compulsory EFL learning course for second-year non-English major college students in China and used the Top-Notch College English integrated course book 3, edition 2. The teaching content comprised reading, listening, and some translation exercises. All 50 students took the course in all three modes for this study, conducting ten 50-min course sessions per mode. The teacher initiated a specific number of interactions during each session and waited up to around 20s for student responses after each question. The questions were set at a slightly challenging level for the average students. The difficulty may have varied from session to session; however, this variation was minimized since the results were based on the participants’ overall perception of the 10 sessions in each mode.

#### 4.2.2. Normal classroom mode

In this mode, students engaged in traditional face-to-face communication without using any SCMC technology. They were allowed to access the internet through their personal smartphones during the course. This mode served as the control group and blank sample, providing a baseline for comparison with other modes and representing a typical teaching scenario in daily practice.

#### 4.2.3. Pure SCMC mode

In this mode, students participated in the course through live webcasts and engaged in text-or voice-based computer-mediated communication (SCMC). Traditional face-to-face communication was not present, and the course was conducted solely through SCMC technology.

#### 4.2.4. Hybrid SCMC (BYOD) mode

In this mode, students were in an FTF classroom and simultaneously logged into the live classroom on the internet through their smartphones and open internet (BYOD) to participate in the interaction. This allowed the students to interact either through text-based SCMC or in the same manner as in a traditional FTF classroom.

#### 4.2.5. FLCAS questionnaire

Students were requested to take the FLCAS survey for each mode after completing the learning activity. A complete questionnaire that was used to measure students’ FLCAS is provided in Appendix I (33 items). In addition, the results of 18 items (which this study considers more relevant to classroom interaction and marked with “*” in Appendix I) were selected as the FLCAS1 section for analysis to more closely observe the FLCAS change related to classroom interaction.

#### 4.2.6. Perceptual questionnaire

In addition, students were requested to participate in perceptual questionnaire surveys upon completion of their courses (see Appendix II). Based on their perception, they were asked to vote on their feelings about the three factors (anxiety, distraction from the internet, and classroom atmosphere) in the three modes (questions 1–5). In addition, they were asked to score the interaction efficiency of the three modes (question 6).

#### 4.2.7. Open responses

After finishing their perception questionnaire, students were also asked to provide an open response to explain why they voted for the three factors in that way, based on their perception. This is to understand the reasons and feelings behind their vote choices. Since some participants might not have been able to explain their reasoning, the open-ended response was not compulsory.

### 4.3. Procedure

All 50 students first took 10 sessions of the College English course in pure SCMC mode during the quarantine period at the beginning of the semester; then, when the quarantine was over, they took 10 sessions of the course in FTF mode and subsequently 10 sessions in hybrid SCMC (BYOD) mode. The procedure was performed in this manner because this was a study performed in real teaching practice; thus, it was limited by the university’s schedule and strict quarantine control.

**Figure 1:**
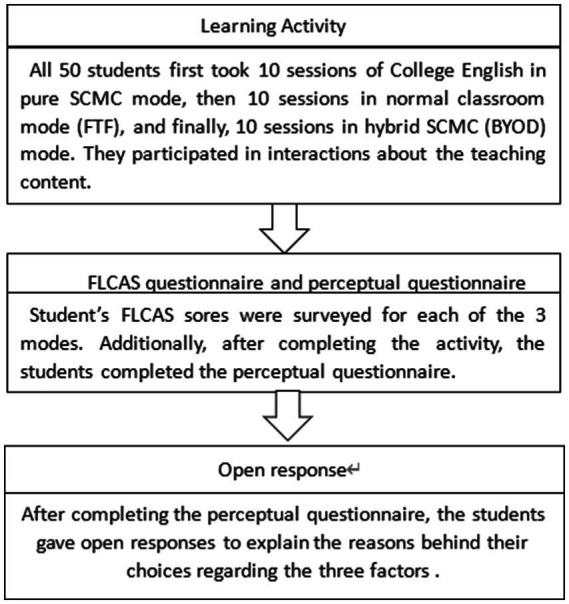
The procedure of the activity and surveys.

FLCAS scores were surveyed for each mode after completing the learning activity. In addition, after completing all three modes, the students were asked to complete the perceptual questionnaires to compare their interaction experiences and the three factors within the three modes. Then, the students provided open responses to explain the reasons behind their choices regarding the three factors in the perceptual questionnaire (see Figure 1).

### 4.4. Data analysis

The data collected from the questionnaire survey were processed as figures and tables. Data on the major factors in the three modes were calculated and processed to determine how these factors relate and change among the three modes. Paired sample t tests were used to analyse the students’ FLA and interaction scores between the blank sample mode and the two SCMC modes. An alpha level of 0.05 was set for the tests. The statistical software used was Microsoft Excel version 2,207.

## 5. Results

The study results showed that students reported better interaction and learning experiences in the hybrid SCMC (BYOD) mode than at baseline [blank sample normal classroom mode (FTF)]. However, no improvement was reported in the pure SCMC mode. The three factors observed using the FLCAS test, perceptual questionnaire, and open responses showed similarities and differences between the two SCMC treatment modes.

The FLCAS test and perceptual questionnaire indicated that both SCMC treatment modes reduced FLA compared to the blank sample, and students’ open responses explained the reason for anxiety reduction. However, the perceptual questionnaire and open response showed that the pure SCMC mode worsened the classroom atmosphere, while the hybrid SCMC (BYOD) mode improved it. Both SCMC treatments also led to an increase in internet distraction, but the increase was much smaller in the hybrid SCMC (BYOD) mode.

### 5.1. Students’ perceptual score assessment of the degree of interaction participation

Students were instructed to score their degree of interaction participation in the three modes (see Appendix 2, question 6). The maximum score was 5 (Table 1).

**Table 1:** The degree of interaction participation scored by students (Range 0–5).

	Mean	Mdn	SD	Total score
Normal classroom mode (FTF)	3.52	4	1.04	176
Pure SCMC mode (Live Webcast)	3.36	3	0.82	168
Hybrid SCMC (BYOD) mode (SCMC-Assisted FTF Classroom)	4.28	4.5	0.82	214

**Figure 2:**
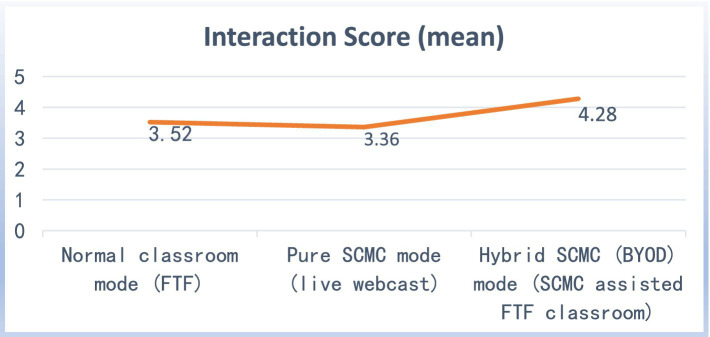
Student scores of the degree of interactive participation.

The students’ perceptions of interaction participation in the three modes are presented in the interaction score comparison chart (Figure 2). The survey results show that students rated the hybrid SCMC (BYOD) mode the highest, followed by the normal classroom mode and the pure SCMC mode. According to the students’ perception, the survey revealed that the pure SCMC mode did not significantly impact (*t* = 0.91, *p* > 0.05) the students’ interaction and learning experience in comparison with the blank sample normal FTF mode. However, there was a significant improvement in the hybrid SCMC (BYOD) mode (*t* = −4.26, *p* < 0.05).

### 5.2. FLA changes

The students’ FLA was measured using the FLCAS questionnaires (33 items; Appendix I) in each of the 3 modes. Furthermore, questions 1–3 in the perceptual questionnaires (see Appendix II) were conducted to find whether they felt obvious anxiety that hindered their interaction.

#### 5.2.1. FLCAS survey

FLCAS questionnaires (33 items) were used to measure the students’ FLA in each of the three modes. This study selected the results of 18 items (which the author considers most relevant to classroom interaction and marked with “*” in Appendix I) as the FLCAS1 section to more closely observe the FLCAS change related to classroom interaction while excluding replies to items such as “item 10: I worry about the consequences of failing my foreign language class. Item 30: I feel overwhelmed by the number of rules you have to learn to speak a foreign language,” which are considered less relevant to class interaction (see Table 2).

**Table 2:** FLCAS results (full 33 items in Appendix 1).

FLCAS (33 items)	Mean	Mdn	SD
Normal classroom mode (FTF)	109.48	111	18.48
Pure SCMC mode (Live webcast)	89.74	89	17.34
Hybrid SCMC (BYOD) mode (SCMC-Assisted FTF classroom)	93.92	94	15.69

##### 5.2.1.1. FLCAS (33 items) results analysis

The target students had low English proficiency, as evidenced by their CET 4 passing rate of 30%. Their FLCAS score was high in the normal face-to-face (FTF) classroom mode, with a mean of 109.48 and a median of 111, which was higher than the average test group score (mean 92.03, median 95) reported by Shao et al. (2013).

The mean FLCAS score in the normal classroom mode (the blank sample) was 109.48. However, when the pure synchronous computer-mediated communication (SCMC) mode was used, the mean FLCAS score significantly decreased to 89.74 (median 89) (*t* = 9.05, *p* < 0.05). Similarly, the mean FLCAS score in the hybrid SCMC (BYOD) mode was 93.92 (median 94), which also showed a significant decrease compared with that in the normal FTF mode (*t* = 9.85, *p* < 0.05) (see Table 2).

##### 5.2.1.2. FLCAS1 (18 items) results analysis

The analysis of FLCAS1, comprising 18 items deemed more relevant to classroom interaction, also revealed a significant reduction in both SCMC and hybrid modes. In the normal classroom mode (FTF, blank sample), the mean FLCAS1 mean score was 59, and the median score was 58. When the pure SCMC mode was implemented, the mean FLCAS1 score of 44.12, and the median score was 41. These scores were significantly lower compared to those in normal classroom mode (FTF) (*t* = 8.12, *p* < 0.05). Similarly, when the hybrid SCMC (BYOD) mode was employed, the mean FLCAS1 score was 47.76, and the median score was 47, also significantly lower than the normal classroom mode (FTF) scores (*t* = 8.88, *p* < 0.05). (see Table 3).

**Table 3:** FLCAS1 results (18 items with* in Appendix 1).

FLCAS1 (18 items)	Mean	Mdn	SD
Normal classroom mode (FTF)	59	58	13.48
Pure SCMC Mode (Live webcast)	44.12	41	12.54
Hybrid SCMC (BYOD) mode (SCMC-Assisted FTF classroom)	47.76	47	11.05

#### 5.2.2. Obvious anxiety felt

Questions 1–3 in the perceptual questionnaire (found in Appendix 2) asked about students’ shyness, anxiety, and fear that hindered their interaction during the three observation modes. The results are recorded in Table 4, and a negative value was assigned to the number of responses of “Yes” for shyness, anxiety, and fear, as these emotions negatively impact classroom interaction.

**Table 4:** Obvious anxiety felt.

	Obvious anxiety felt (votes from 50 students)	FLCAS 1(18 items) mean
Normal classroom mode (FTF)	−38	59 (high)
Pure SCMC Mode (Live webcast)	−3 (+35^*^)	44.12 (low)
Hybrid SCMC (BYOD) mode (SCMC-Assisted FTF classroom)	−6 (+32^*^)	47.76 (low)

“+ number *” indicates a positive change in the learning experience and interaction compared to the blank sample mode, while “- number *” indicates a negative change in the learning experience and interaction compared to the blank sample mode.

Of the 50 students, 38 reported obvious anxiety during normal classroom mode (FTF) interaction, whereas only 3 reported obvious anxiety during pure SCMC mode interaction, and 6 during hybrid SCMC (BYOD) mode interaction. This trend is consistent with the findings of the FLCAS1 test (Section 5.21). The anxiety votes for normal classroom mode (FTF) interaction were assigned A0 = –38, for pure SCMC mode interaction A1 = –3, and for hybrid mode interaction A2 = -6. Compared to the normal classroom mode (FTF), the anxiety votes were reduced by +35*(A1-A0) in the pure SCMC mode and by +32*(A2-A0) in the hybrid mode.

Although the pure SCMC and hybrid SCMC (BYOD) modes showed a positive effect on reducing anxiety, there was a significant difference in their overall effect on interaction and learning experience, as shown in section 5.1. The pure SCMC mode did not show any improvement in interaction compared to the normal classroom mode (FTF), while there was a considerable improvement in the hybrid mode. This finding suggests that other factors may be responsible for the difference in interaction between the two modes and that the difference in anxiety reduction may not be the sole cause of the difference in interaction.

##### 5.2.2.1. Students’ responses explaining their vote difference for anxiety

According to the questionnaire, 12 and 6% of the 50 students reported experiencing significant anxiety that hindered their interaction in the pure SCMC (live webcast) and hybrid modes, respectively. In contrast, 76% of students reported feeling significant anxiety in the traditional classroom mode. Subsequently, an open response question was posed to students who reported noticeable anxiety in the traditional classroom mode but not in SCMC modes. Based on their perceptions, these students were asked to briefly explain why they did not feel significant anxiety when communicating in pure SCMC mode and hybrid SCMC (BYOD) mode, as they did in the traditional face-to-face mode. Additionally, students with alternative perspectives on the topic were encouraged to share their thoughts.

The most representative answers (translated from Chinese, edited and summarized) are as follows:

1. Expressing on the internet was much less stressful and easier for me, because in that way I would not feel the heavy attention and pressure of teachers and peers in the classroom.
2. When answering on internet, I felt that I was more that ID (avatar). Thus, if I made mistakes, it would not embarrass me too much.
3. When discussing on the internet, many students speak at the same time, so I felt less pressure and that I would draw less attention if I made mistakes.
4. Typing English on the internet could be edited and modified, so I made fewer mistakes.
5. The reduction in face-to-face communication could reduce the pressure on students who did not speak much. My shy and conservative classmates were frightened to say anything when facing a room full of people, but they could talk freely when facing a mobile phone.

Response 2 echoes the safety-inducing effect of avatars proposed by Zhao and Lai (2009). Response 4 echoes Charle Poza’s (2005) finding that editable answers in SCMC may help reduce FLA. However, his finding related to editing voice answers in Voice-SCMC, while this response concerns editing text answers.

In summary, anonymity, editable answers, shyness, conservative personality, and less attention from others were the major reasons students preferred to express themselves through SCMC methods.

### 5.3. Distraction from the internet

Question 4 in the perceptual questionnaire (found in Appendix 2) assessed whether students easily become distracted by internet-related factors, such as games and social software. As these distractions have a negative impact on classroom interaction, this study assigned a negative value for the number of “Yes” votes (as seen in Table 5).

**Table 5:** Distraction from the internet.

	Distractions (votes from 50 students)
Normal classroom mode (FTF)	−17
Pure SCMC mode (Live webcast)	−41 (−23^*^)
Hybrid SCMC (BYOD) mode (SCMC-Assisted FTF classroom)	−24(−7^*^)

“+ number *” indicates a positive change in the learning experience and interaction compared to the blank sample mode, while “- number *” indicates a negative change in the learning experience and interaction compared to the blank sample mode.

#### 5.3.1. Survey results for distraction from the internet

The distraction from internet votes for the normal classroom mode (FTF) were assigned D0 = –17, for the pure SCMC mode D1 = –41, and for the hybrid SCMC (BYOD) mode D2 = –24. Compared to the normal classroom mode (FTF), distraction votes were increased by −24* (D1-D0) in the pure SCMC mode and by −7* (D2-D0) in the hybrid SCMC (BYOD) mode. Both SCMC modes showed a higher level of distraction from the internet compared to the normal classroom mode (FTF), but the increase was less pronounced in the hybrid SCMC (BYOD) mode.

#### 5.3.2. Students’ responses on explaining their vote difference for distraction from internet

According to the questionnaire, 34 and 48% of 50 students reported that they were easily distracted in the traditional classroom and hybrid modes, respectively. In contrast, 82% of students indicated that they were easily distracted in the pure SCMC mode. Subsequently, an open response question was posed to students who reported being easily distracted by the internet in pure SCMC mode but not in the other two modes. Based on their perceptions, these students were asked to briefly explain why they felt more easily distracted in the pure SCMC mode compared to the other modes. Additionally, students with alternative perspectives on the topic were encouraged to share their thoughts.

The most representative responses (translated from Chinese, edited and summarized) are as follows:

1. In the same room with teacher and other students, I felt I was more supervised. In a real classroom, I felt that I was restrained from using online entertainment.
2. In a pure online classroom, since I used smartphone and internet for the class, I was easily tempted to play with my smartphone from time to time. However, in a face-to-face classroom, if I played with my smartphone, I feared I would be easily noticed by the teacher and other students.
3. In an FTF classroom, I felt I was more immersed in the class. In addition, due to the learning habit formed in many years, I dared not play with my smartphone in front of teachers and other students. However, in the pure SCMC mode, I dared to play with my smartphone.
4. In the pure SCMC mode, I was easily tempted by smartphone and internet entertainment. The FTF classroom provided a more academic and immersive atmosphere, and I would feel ashamed to play games while other students around me were studying.
5. In the pure SCMC mode, the teacher could not effectively watch over me, so I could do whatever I wanted.
6. In the hybrid SCMC (BYOD) and FTF modes, in an FTF classroom, when I saw my good friends sitting there, I felt tempted to chat with them through voice or smartphone chat software, so I was more distracted. However, when alone at home in the pure SCMC mode, I did not have such interest or urge.

In summary, those who felt modes involving the FTF classroom were less distracting mainly reflected reasons including teacher and students’ supervision, immersive environment, and formed learning habits. However, there were also students who had the opposite perception that modes with an FTF classroom environment might cause more distraction from the internet, as response 6 stated.

### 5.4. Classroom atmosphere

Question 5 in the perceptual questionnaire (found in Appendix 2) assessed students’ perceptions of the liveliness of the classroom atmosphere during the course. A positive value assigned to the votes since a lively atmosphere is believed to positively impact classroom interaction (Table 6).

**Table 6:** Classroom atmosphere.

	Good class atmosphere felt (votes from 50 students)
Normal Classroom Mode (FTF)	24
Pure SCMC Mode (Live Webcast)	8(−16*)
Hybrid SCMC (BYOD) Mode (SCMC-Assisted FTF Classroom)	39(+15*)

“+ number *” indicates a positive change in the learning experience and interaction compared to the blank sample mode, while “- number *” indicates a negative change in the learning experience and interaction compared to the blank sample mode.

The results showed that in the normal classroom mode (FTF), 24 students reported a good atmosphere, while 8 students reported it in pure SCMC mode and 39 in the hybrid SCMC (BYOD) mode. Compared to the blank sample mode, the pure SCMC mode showed a decrease of −16* in the good classroom atmosphere, while the hybrid SCMC (BYOD) mode showed an increase of +15*. This suggests that while the pure SCMC mode had a negative impact on the classroom atmosphere, the hybrid mode had a positive impact (see Table 7).

**Table 7:** Comparison among three modes.

	Obvious anxiety felt (votes from 50 students)	Distractions from the internet (votes from 50 students)	Good class atmosphere (votes from 50 students)
Normal Classroom Mode (FTF, Blank Sample)	−38	−17	24
Pure SCMC Mode (Live Webcast)	−3 (+35* positive change compared to FTF mode blank sample)	−41 (−24* negative change)	8 (−16* negative change)
Hybrid Mode (SCMC-Assisted FTF Classroom)	−6 (+32* positive change)	−24 (−7* negative change)	36 (+12* positive change)

“+ number *” indicates a positive change in the learning experience and interaction compared to the blank sample mode, while “- number *” indicates a negative change in the learning experience and interaction compared to the blank sample mode.

#### 5.4.1. Students’ responses explaining their vote difference for good classroom atmosphere

According to the questionnaire, 48% of the 50 students reported a positive classroom atmosphere in the traditional classroom mode, while 78% reported the same in the hybrid mode. In contrast, only 16% of students felt a good classroom atmosphere in the pure SCMC mode. Subsequently, an open response question was posed to students who reported a positive atmosphere in traditional and hybrid classroom modes but not in pure SCMC modes. Based on their perceptions, students were asked to briefly explain why they felt the atmosphere was good in traditional and hybrid classrooms but not in the pure SCMC classroom. Additionally, students with alternative perspectives on the topic were encouraged to share their thoughts.

The most representative responses (translated from Chinese, edited and summarized) are as follows:

1. The FTF classroom condition and the hybrid mode provided more variety and convenience for communication; thus, the atmosphere was livelier.
2. In the pure SCMC mode, due to technical issues and lagging, we could not see other students and teachers in the way we could in a face-to-face classroom. Voice communication was also not smooth due to network latency.
3. I could not feel the mood of other students in the pure SCMC mode.
4. In the FTF and hybrid SCMC (BYOD) modes, I sat in an FTF classroom; thus, I felt I was more intimate with other students and teacher and gesture communication was possible, while the pure SCMC mode of communication gave a cold feeling, and voice communication was not smooth due to network latency.
5. In the normal classroom FTF mode, I was not involved much in interaction due to shyness. However, in the hybrid SCMC (BYOD) mode, when online interaction was added in the FTF classroom, I would participate in the discussion, as would many others. As a result, we were motivated by each other, and the atmosphere became better.

Possible gesture communication in the hybrid SCMC (BYOD) mode mentioned in response 4 echoes York et al.’s (2021) supposition that paralinguistic cues such as gesture communication may help to improve the learning experience.

In summary, the responses showed that the modes with face-to-face (FTF) classroom settings had a better atmosphere, while pure online SCMC communication provided a cold atmosphere and had limited means of communication. Technical difficulties and network latency also had a negative effect on the atmosphere in a purely online setting. In contrast, the hybrid SCMC approach, which combines FTF and online elements, reduced shyness among students, resulting in improved participation and atmosphere.

### 5.5. Group comparison of the factors among the three modes

The pure SCMC mode showed negative changes in the learning experience and interaction compared to the FTF mode blank sample. The votes for anxiety (FLA) were reduced by +35*, but the votes for internet distraction increased by −24*, and the votes for good class atmosphere decreased by −16*. Although there was an improvement in the reduction of anxiety, the increase in distraction and decline in class atmosphere had a negative impact on the overall experience.

In comparison to the FTF mode blank sample, the hybrid SCMC (BYOD) mode showed a decrease in anxiety (FLA) by +32* and an improvement in good class atmosphere by +12*. There was also a slight increase in internet distraction by −7*. The reduction in anxiety, similar to that in pure SCMC, and the improvement in classroom atmosphere positively impacted the overall learning experience. Despite the small increase in internet distraction, it was less pronounced than that in the pure SCMC mode.

The comparison between pure SCMC and hybrid SCMC (BYOD) modes indicates that while both modes effectively reduced anxiety, pure SCMC had a negative impact on the class atmosphere and increased distraction from the internet. This explains why the interaction and learning experience was not improved in the pure SCMC mode (refer to section 5.1).

On the other hand, the hybrid SCMC (BYOD) mode not only reduced anxiety but also improved the class atmosphere, positively influencing the overall experience. Although there was a slight increase in internet distraction, this increase was less significant compared to that observed with pure SCMC. This helps explain why the interaction and learning experience were reported to be improved in the hybrid SCMC (BYOD) mode. (refer to section 5.1).

## 6. Discussion

This study aimed to investigate the impact of the hybrid SCMC (BYOD) mode on FLA, interaction, and learning experiences in comparison to the blank sample mode of the FTF mode (the traditional face-to-face classroom) and the pure SCMC mode. The research adopted a more realistic setting where students utilized their own devices, such as smartphones and PCs, along with open internet access within the classroom, as opposed to a controlled laboratory environment.

The results from the FLCAS test and perception surveys revealed a significant decrease in FLA for both hybrid SCMC (BYOD) mode and pure SCMC mode when compared to the blank sample of normal FTF mode. Open-ended responses indicated that anonymity and the ability to edit answers contributed to the effectiveness of SCMC in reducing FLA, consistent with the findings of Zhao and Lai (2009) and Charle Poza (2005). Furthermore, open responses suggested that some students’ preference for expressing themselves through SCMC could be attributed to their more introverted personalities.

These findings align with previous laboratory-based SCMC studies, demonstrating that both pure SCMC and hybrid SCMC can significantly reduce FLA (Charle Poza, 2005; Satar and Özdener, 2008; York et al., 2021). The key difference in this study is that the results were derived from a typical teaching environment, suggesting that implementing SCMC to reduce FLA could be a practical approach in everyday classroom settings. The challenge arising from this finding is determining how to effectively apply this approach in standard educational conditions.

Concerns have been raised about the lack of supervision, increased distractions, and poor teaching atmosphere in pure SCMC classes during the COVID-19 quarantine (Feng, 2020; Fu, 2020; Jin, 2020; Qu et al., 2021). This study hypothesized that the hybrid SCMC (BYOD) mode (SCMC-assisted FTF classroom) could enhance interaction and learning experiences by reducing FLA while maintaining supervision and a lively atmosphere of the FTF mode. The results largely support this hypothesis, with some differences.

Students’ perception scores showed that the hybrid SCMC (BYOD) mode significantly improved interaction and learning experiences compared to the blank sample of normal FTF mode, while pure SCMC offered no improvement. These findings align with York et al.’s (2021) study on hybrid SCMC using virtual reality (VR), which indicated that hybrid SCMC (VR) delivers a better learning experience than pure SCMC. However, the factors contributing to improved learning experiences differ. Although both studies acknowledged more realistic and immersive environments in hybrid modes, York’s study attributed improvements to curiosity about VR, while the hybrid SCMC (BYOD) mode credited less increased internet distraction and an improved classroom atmosphere.

The results concerning internet distractions and classroom atmosphere indicated that pure SCMC negatively impacted both factors compared to the blank sample of normal FTF mode. The hybrid SCMC (BYOD) mode did not fully align with the hypothesis. On the one hand, hybrid SCMC (BYOD) mode not only maintained but also enhanced the lively atmosphere of the FTF mode, possibly due to reduced shyness among students. On the other hand, although it maintained the supervision of the FTF mode, the hybrid SCMC (BYOD) was still associated with slightly increased distractions from the internet versus the normal FTF mode, likely due to increased smartphone and internet usage. Therefore, one possible approach to improve this SCMC (BYOD) method is to find ways to further minimize this increase in internet distraction.

Open-ended responses elucidated the reasons for enhanced interaction and learning experiences in the hybrid SCMC (BYOD) mode, including increased supervision, immersive experiences, established learning habits, communication methods, and less interference from technical issues and network latency. These insights help us better understand the factors contributing to the improved experience with the hybrid SCMC (BYOD) mode and provide guidance for further refinement.

### 6.1. Significance of the study

This study provides a unique perspective on the impact of SCMC on reducing FLA and enhancing the learning experience, distinguishing itself from previous laboratory-based studies by conducting research mostly in typical classroom settings for more practical and meaningful outcomes. Additionally, this study went beyond examining the effect of SCMC on FLA and analysed other affective factors affecting the learning experience, offering a new perspective on why hybrid SCMC is a better approach for reducing anxiety and enhancing the learning experience.

The authors believe that hybrid SCMC should be researched further due to its potential to reduce FLA through the use of SCMC technology while also providing an immersive environment similar to a face-to-face classroom, including immersive experience, paralinguistic cues, and social presence. Unlike previous studies on VR or virtual worlds, this study applied a hybrid SCMC (BYOD) approach in a real-world classroom setting using students’ smartphones and open internet, making it an inexpensive and practical method for implementation. The findings of this study may provide useful insights into how to improve the practical and inexpensive hybrid SCMC approach, such as by adding mobile device management (MDM).

This study highlights the need for further research on hybrid SCMC (BYOD) modes to reduce FLA, specifically in the classroom setting. In the future, researchers should consider multiple affective factors, beyond just FLA, when examining the impact on learning. Failing to do so could result in reduced FLA but worsen other affective factors, leading to a lack of improvement or even a decline in learning experience and interaction. This is a common issue seen in many online teaching practices.

### 6.2. Implications for future research

The hybrid SCMC (BYOD) mode, being classroom-based and relying on open internet and students’ mobile phones, may still result in internet distractions. To address this issue, researchers should consider incorporating mobile device management (MDM) technology. This technology, which originated from the BYOD concept in workplaces, allows for controlled access to internet resources on employees’ mobile devices. Similarly, students’ devices in a hybrid SCMC classroom could be managed and supervised effectively. Future research should test the effectiveness of incorporating MDM technology in the hybrid SCMC (BYOD) mode to enhance supervision and improve the overall learning experience.

### 6.3. Study limitations

This study has several limitations. First, we only evaluated three major factors (FLA, internet distraction, and classroom atmosphere) mentioned in previous literature, and there could be other important affective factors that should be accounted for. Second, we used simple perceptual votes and open responses to assess factors such as internet distraction and classroom atmosphere due to lack of funds, researchers and facility support. This method is easy to implement but lacks precision and detail, potentially leading to inaccuracies in the results. Future studies should consider using more established and accurate scales and more detailed questionnaires to evaluate these factors. For example, a questionnaire comprising more than 5 Likert-scale questions for each factor and a t test to check for significance in the results would provide more accurate results. Third, the learning procedure was limited by the teaching schedule and quarantine control of the author’s university, as the study was conducted in real teaching practice. Using a 3×3 counterbalanced measure design would have been better to eliminate any potential sequence effect. Finally, the study was limited to one university due to funding and support constraints, and it would be beneficial to conduct further research in multiple universities with more participants.

## 7. Conclusion

The results of this study suggest that the hybrid SCMC (BYOD) mode, which combines SCMC and face-to-face teaching, reduces the students’ anxiety level and results in better interaction and learning experiences than traditional face-to-face teaching and pure SCMC. The FLCAS results showed significant decreases in anxiety levels in both hybrid SCMC and pure SCMC modes, and the results of the perceptual questionnaires were consistent with this trend, with students reporting better interaction and learning experience in the hybrid SCMC (BYOD) mode.

This improvement in interaction and learning experience in the hybrid SCMC (BYOD) mode is likely due to a combination of factors, including lower anxiety levels, better supervision, and a livelier classroom atmosphere. The students’ open responses revealed several detailed reasons behind the improved interaction and learning experiences in the hybrid SCMC (BYOD) mode, offering valuable insights for its ongoing refinement.

This study emphasizes the potential of hybrid SCMC in reducing FLA and enhancing learning experiences within real-world classroom settings while considering multiple affective factors. The practical and cost-effective nature of the hybrid SCMC (BYOD) approach using students’ smartphones and open internet access underscores the need for further research in this area to optimize its implementation and impact on learning.

## Data availability statement

The original contributions presented in the study are included in the article/Supplementary material, further inquiries can be directed to the corresponding author.

## Ethics statement

Ethical review and approval was not required for the study on human participants in accordance with the local legislation and institutional requirements. Written informed consent for participation was not required for this study in accordance with the national legislation and the institutional requirements.

## Author contributions

The author confirms being the sole contributor of this work and has approved it for publication.

## Conflict of interest

The author declares that the research was conducted in the absence of any commercial or financial relationships that could be construed as a potential conflict of interest.

## Publisher’s note

All claims expressed in this article are solely those of the authors and do not necessarily represent those of their affiliated organizations, or those of the publisher, the editors and the reviewers. Any product that may be evaluated in this article, or claim that may be made by its manufacturer, is not guaranteed or endorsed by the publisher.

## Supplementary material

The Supplementary material for this article can be found online at: https://www.frontiersin.org/articles/10.3389/fpsyg.2023.1172442/full#supplementary-material
