# Supplementary material for: The effect of hybrid SCMC (BYOD) on foreign language anxiety and learning experience in comparison to pure SCMC and FTF communication
Source: Front Psychol. 2023 Jul 25;14:1172442. doi: 10.3389/fpsyg.2023.1172442 (PMC10433158; doi:10.3389/fpsyg.2023.1172442)
Supplement: Supplementary file 1 [file Data_Sheet_1.pdf]

Authors: Elaine K. Horwitz, Michael B. Horwitz, and Joann Cope

Article: Foreign Language Classroom Anxiety

Journal: The Modern Language Journal

Publisher: John Wiley & Sons, Inc

Copyright notice: ©1986 *The Modern Language Journal*

Reproduced with permission from 'Foreign Language Classroom Anxiety', Elaine K. Horwitz, Michael B. Horwitz, and Joann Cope, published in The Modern Language Journal, 70, 125-132, 1986, by John Wiley & Sons, Inc., under license No. 5571420133254, with additional approval from the Wiley Permission Group to modify the source material.

Appendix I

The Foreign Language Classroom Anxiety Scale (FLCAS)

Score range (1-5): 1 strongly disagree, 2 disagree, 3 undecided, 4 agree, 5 strongly agree

1*. I never feel quite sure of myself when I am speaking in my foreign language class.

2*. I don't worry about making mistakes in language class.

3*. I tremble when I know that I'm going to be called on in language class.

4*. It frightens me when I don't understand what the teacher is saying in the foreign language.

5. It wouldn't bother me at all to take more foreign language classes.

6. During language class, I find myself thinking about things that have nothing to do with the course.

7. I keep thinking that the other students are better at languages than I am.

8*. I am usually at ease during tests in my language class.

9*. I start to panic when I have to speak without preparation in language class.

10. I worry about the consequences of failing my foreign language class.

11. I don't understand why some people get so upset over foreign language classes.

12*. In language class, I can get so nervous I forget things I know.

13*. It embarrasses me to volunteer answers in my language class.

14.I would not be nervous speaking the foreign language with native speakers.

15. I get upset when I don't understand what the teacher is correcting.

16*. Even if I am well prepared for language class, I feel anxious about it.

17. I often feel like not going to my language class.

18*. I feel confident when I speak in foreign language class.

19*. I am afraid that my language teacher is ready to correct every mistake I make.

20*. I can feel my heart pounding when I'm going to be called on in language class.

21.The more I study for a language test, the more confused I get.

22. I don't feel pressure to prepare very well for language class.

23. I always feel that the other students speak the foreign language better than I do.

24*. I feel very self-conscious about speaking the foreign language in front of other students.

25. Language class moves so quickly I worry about getting left behind.

26*. I feel more tense and nervous in my language class than in my other classes.

27*. I get nervous and confused when I am speaking in my language class.

28. When I'm on my way to language class, I feel very sure and relaxed.

29*. I get nervous when I don't understand every word the language teacher says.

30. I feel overwhelmed by the number of rules you have to learn to speak a foreign language.

31*. I am afraid that the other students will laugh at me when I speak the foreign language.

32. I would probably feel comfortable around native speakers of the foreign language.

33*. I get nervous when the language teacher asks questions which I haven't prepared in advance.
