# Supplementary material for: The effect of hybrid SCMC (BYOD) on foreign language anxiety and learning experience in comparison to pure SCMC and FTF communication
Source: Front Psychol. 2023 Jul 25;14:1172442. doi: 10.3389/fpsyg.2023.1172442 (PMC10433158; doi:10.3389/fpsyg.2023.1172442)
Supplement: Supplementary file 2 [file Data_Sheet_2.pdf]

Appendix II

1 When you were interacting with teachers and other students in an English language course (Normal classroom mode), did you experience obvious shyness characterized by fear or anxiety when communicating; or obvious "apprehension about others' evaluation and the expectation that others would evaluate you negatively." Y/N

2 When you were interacting with teachers and other students in an English language course (pure SCMC mode, live webcast), did you experience obvious shyness characterized by fear or anxiety when communicating; or obvious "apprehension about others' evaluation and the expectation that others would evaluate you negatively." Y/N

3 When you were interacting with teachers and other students in English language course (Hybrid mode, SCMC assisted FTF classroom), did you experience obvious shyness characterized by fear or anxiety when communicating; or obvious "apprehension about others' evaluation and the expectation that others would evaluate you negatively." Y/N

4 The distractions of Web and social software (tick on the statement that is true for you, (you can choose multiple statements):

A. I am easily distracted by web and social software in normal classroom mode (FTF)

B. I am easily distracted by web and social software in SCMC mode (live webcast)

C. I am easily distracted by web and social software in a hybrid mode (SCMC assisted FTF classroom)

5 Which of the following mode has good class atmosphere? (You can choose multiple options)

A. Normal classroom mode (FTF)

B. SCMC mode (live webcast)

C. Hybrid mode (SCMC assisted FTF classroom)

6 If you are asked to give a score to evaluate the degree of interaction participation under three modes, what score would you give? (The full score is 5)

Normal classroom mode (FTF) _____

SCMC mode (live webcast) _____

Hybrid mode (SCMC assisted FTF classroom) _____
